# Studies on the non-invasive anticancer remedy of the triple combination of epigallocatechin gallate, pulsed electric field, and ultrasound

**DOI:** 10.1371/journal.pone.0201920

**Published:** 2018-08-06

**Authors:** Chih-Hsiung Hsieh, Chueh-Hsuan Lu, Yu-Yi Kuo, Wei-Ting Chen, Chih-Yu Chao

**Affiliations:** 1 Department of Physics, Lab for Medical Physics & Biomedical Engineering, National Taiwan University, Taipei, Taiwan; 2 Biomedical & Molecular Imaging Center, National Taiwan University College of Medicine, Taipei, Taiwan; 3 Institute of Applied Physics, National Taiwan University, Taipei, Taiwan; Institute of Biochemistry and Biotechnology, TAIWAN

## Abstract

Cancer is one of the most troublesome diseases and a leading cause of death worldwide. Recently, novel treatments have been continuously developed to improve the disadvantages of conventional therapies, such as prodigious expenses, unwanted side effects, and tumor recurrence. Here, we provide the first non-invasive treatment that has combined epigallocatechin gallate (EGCG), the most abundant catechin in green tea, with a low strength pulsed electric field (PEF) and a low energy ultrasound (US). It has been observed that the cell viability of human pancreatic cancer PANC-1 was decreased approximately to 20% of the control after this combination treatment for 72 h. Besides, the combined triple treatment significantly reduced the high tolerance of HepG2 cells to the EGCG-induced cytotoxicity and similarly exhibited compelling proliferation-inhibitory effects. We also found the combined triple treatment increased the intracellular reactive oxygen species (ROS) and acidic vesicles, and the EGCG-induced inhibition of Akt phosphorylation was dramatically intensified. In this study, the apoptosis inhibitor Z-VAD-FMK and the autophagy inhibitor 3-MA were, respectively, shown to attenuate the anticancer effects of the triple treatment. This indicates that the triple treatment-induced autophagy was switched from cytoprotective to cytotoxic, and hence, cooperatively caused cell death with the apoptosis. Since the EGCG is easily accessible from the green tea and mild for a long-term treatment, and the non-invasive physical stimulations could be modified to focus on a specific location, this combined triple treatment may serve as a promising strategy for anticancer therapy.

## Introduction

Cancer is the second leading cause of death worldwide and remains a major challenge for public health research [1]. Traditional therapies such as surgery, radiation, and chemotherapy are commonly used to treat patients diagnosed with this disease. However, patients treated with conventional treatments still have a high risk of tumor recurrence, and many of them are refractory to treatment. Thus, newer approaches to improve the efficiency of a cancer therapy at an affordable cost are urgently needed. The most common methods are combination therapies that employ two or more anticancer drugs, and these strategies are considered to target different pathways and to enhance their therapeutic effectiveness in a synergistic or additive manner [2]. Nevertheless, combination therapies could also reduce efficacy due to the drug competition [3]. Besides, unwanted side effects and dangerous drug interactions still exist as the potentially harmful effects.

Recently, we have reported that a non-invasive low strength pulsed electric field (PEF) can enhance the epigallocatechin gallate (EGCG) to combat against the pancreatic cancer cells [4]. It was found that the synergistic reactions in the double treatment of the EGCG and PEF disturbed the mitochondria, enhanced the intrinsic pathway transduction, and effectively induced apoptosis. On the other hand, it has been reported that EGCG is not stable and simultaneously transforms into many EGCG auto-oxidation products (EAOPs) in the cell culture system [5, 6]. Even so, one of the EAOPs, theasinensin A, has also been shown to cause apoptotic cell death in cancer cells [7]. Recently, certain EAOPs have been demonstrated to possess equivalent cytotoxic activities as EGCG and to exhibit an enhanced ability to deplete sulfhydryl group of cysteine, which is a major source for sustaining cancer cell malignancy [6]. Therefore, we suggested the natural products of EGCG combined with the non-invasive and moderate physical stimulations would be a promising strategy in the anticancer treatment.

In addition to the non-invasive PEF, an alternative application of physical stimulations is the exposure of cancer cells to the ultrasound (US), and this technique has been developed not only as a diagnostic imaging modality, but also as a focal therapy modality. High intensity focused ultrasound (HIFU) has been achieved as a tumor ablation technology, and its successful clinical study has been carried out for over a decade [8, 9]. The major mechanisms of the HIFU are mechanical and thermal effects due to the violent acoustic cavitation, and the energy absorbed by the tissue raises the temperature rapidly to 60–85°C, causing coagulation of the proteins, fusion of the cell membranes, and necrosis of the tumor cells [10, 11]. However, normal tissues surrounding the tumor may receive irreversible damage because of the thermal effects [8], and an inadequate therapeutic assessment may result in thermal lesion formation. Additionally, this high intensity treatment was reported to induce severe complications in patients with hepatic and pancreatic cancer [12]. On the other hand, a moderate power US combined with the microbubble was developed as a novel emerging treatment. When the medium is applied with US and undergoes a rapid change of pressure, the microbubble goes through the repetitive forced expansion and compression, resulting in the intensified cavitation bubble collapse [13]. It was reported that the enhanced oscillation of the microbubble was utilized to increase the permeability of the cell membrane thereby enhancing the drug and gene delivery [14, 15]. A recent study further showed that the combined treatment of the US and the microbubble could directly induce apoptotic cell death [16]. However, the microbubble has also been reported to cause unwanted side effects, such as myocardial injury [17], headaches [18], and abdominal pain [19]. Moreover, severe immunogenic and allergic reactions could occasionally occur in patients [20]. Therefore, low intensity US [21, 22] in combination with a non-toxic agent or with other non-invasive physical stimulations would be more suitable for clinical therapy.

The focus of this study is to investigate whether the application of the PEF together with the US to the EGCG treatment could synergistically amplify the anticancer effects and thereby exhibit great efficacy in combating various types of cancer. This study, as far as we know, is the first demonstration of a triple treatment combining one herbal administration and two physical stimulations. We found that this non-invasive treatment could overcome the cytotoxicity tolerance of the human hepatoma HepG2 cells to EGCG and significantly reduce the cell viability of both the PANC-1 and the HepG2 cells. It was also observed that the triple treatment increased the intracellular reactive oxygen species (ROS) and acidic vesicles, and both autophagy and apoptosis were activated to cooperatively induce cell death. In this study, we have not only demonstrated that the anticancer treatment combining the EGCG with the physical stimulations was promising with compelling efficacy, but also elucidated the mechanism underlying its synergistic inhibition effects.

## Materials and methods

### Cell culture

The human pancreatic cancer cell line PANC-1, the human hepatocellular carcinoma cell line HepG2, and the human embryonic kidney 293 (HEK293) were obtained from Bioresource Collection and Research Center (BCRC) (Hsinchu, Taiwan), and each STR-PCR profile for cell line authentication has been well recognized at BCRC. Each cell line was cultured in Dulbecco’s modified Eagle’s medium (DMEM) (HyClone, South Logan, UT, USA) supplemented with 10% fetal bovine serum (FBS) (HyClone), 100 unit/ml penicillin (Gibco Life Technologies, Grand Island, NY, USA), and 100 mg/ml streptomycin (Gibco Life Technologies) at 37°C in humidified air with 5% CO_2_. All the cells were harvested with 0.05% trypsin–0.5 mM EDTA solution (Gibco Life Technologies) during exponential growth and prepared for *in vitro* experiments.

### Treatment with the autophagy or apoptosis inhibitors

The PANC-1 cells were seeded into 35-mm-diameter culture dishes (2 × 10^5^ cells/dish) and allowed to adhere overnight. For the autophagy inhibitor analysis, the cells were pretreated with 1 mM 3-Methyladenine (3-MA) (MedChem Express, Monmouth Junction, NJ, USA) for 1 h. For the apoptosis inhibitor analysis, the cells were pretreated with 20 μM Z-VAD-FMK (MedChem Express) for 1 h.

### Preparation of EGCG

The EGCG was purchased from Sigma-Aldrich (St. Louis, MO, USA) and dissolved in distilled water. Since sonication has been shown to improve the quality of antioxidant compounds during processing and storage of a fruit juice [23, 24], dissolving the EGCG in this study was carried out with the help of ultrasonic processing and dispersion (5 s rectangular pulse with 5 s interval, amplitude 50%, by a Q125 sonicator; Qsonica, Newtown, CT, USA) for 1 h at room temperature. Then, the EGCG was instantly stored at −20°C as a stock solution. After treated with or without the inhibitors, the cells were incubated with various concentrations of EGCG alone or combined with further physical stimulations.

### Experimental setup for exposure of the cells to the US

**Fig 1:**
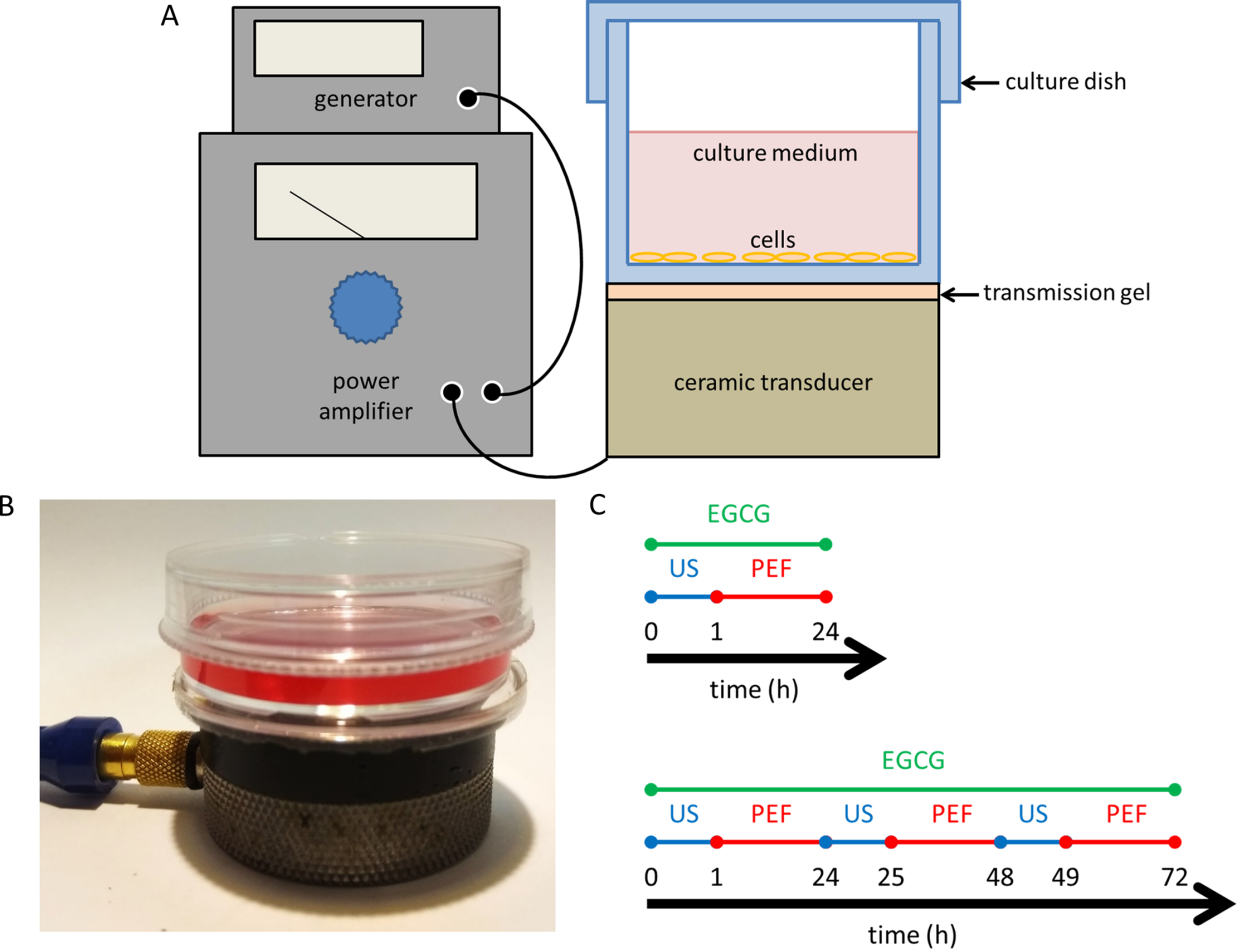
Experimental design of the combined triple treatment. (A)(B) The US exposure system was equipped with a function generator, a power amplifier, and a planar transducer. The 35-mm culture dish was placed on the ceramic transducer, which conducted continuous pulses with an intensity of 0.3 W/cm^2^, pulse period 1 ms, and pulse width 0.5 ms. (C) Experimental schedule of the triple treatment for 24 h (upper) and 72 h (lower).

The US exposure system was equipped with a function generator (SG382; Stanford Research Systems, Sunnyvale, CA, USA), a power amplifier (25a250a; amplifier research, Souderton, PA, USA), and a planar transducer (A104S-RM; Olympus NDT Inc., Waltham, MA, USA). Continuous pulses with amplitude -10 dBm, pulse period 1 ms, and pulse width 0.5 ms were produced using the function generator. As shown in Fig 1A and 1B, the 35-mm culture dish was placed on the ceramic transducer (resonance frequency 2.25 MHz), which converted electrical into acoustic power. To avoid the temperature rise and the undesirable thermal effects induced by the US, the output power of the spatial average intensity of the US exposure was adjusted to be 0.3 W/cm^2^ according to the previous studies [25, 26]. After the EGCG was added to the culture medium, the cells were exposed to different duration of the US (0 to 60 min) for the parameter test. Following, 30 and 60 min of US exposure were employed in the triple treatment for the PANC-1 and HepG2 cells, respectively.

### Experimental setup for exposure of the cells to the PEF

The device for exposure of the cells to the PEF was performed as we previously described [4]. Following the US stimulation, the cells were placed between two copper flat and parallel electrodes. Consecutive pulses with the electric field strength corresponding to 60 V/cm, pulse width 2 ms, and the frequency 2 Hz were applied to the electrodes. The cells treated with continuous exposure of the PEF were kept at 37°C in a humidified atmosphere of 5% CO_2_. The experimental schedule of the combined triple treatments for 24 and 72 h are respectively shown in Fig 1C.

### Cell proliferation assay

3-(4,5-dimethylthiazol-2-yl)-2,5-diphenyltetrazolium bromide (MTT) (Sigma-Aldrich) was dissolved in distilled water to make a 5 mg/ml stock solution. The cell viability of both the PANC-1 and HepG2 cells was measured at 24 h after each treatment. The medium was removed and replaced with a fresh one containing 0.5% mg/ml MTT, and the cells were further incubated for 4 h at 37°C. Following the removal of the supernatant, 2 ml of DMSO was added to each culture dish for dissolving the formazan crystals. The absorbance was measured at 570 nm using Multiskan GO microplate Spectrophotometer (Thermo Scientific, Hudson, NH, USA). Besides, since the cytotoxic activity of EGCG may be underestimated by the MTT method [27], the CellTiter-Glo® luminescent cell viability assay kit was used for validation of the triple treatment-induced anticancer effects. After treatment for 24 h or 72 h, the cells were processed according to the manufacturer's instructions, and the ATP content was determined by reading luminescence using a Flexstation 3 microplate reader (Molecular Devices, Sunnyvale, CA, USA).

### ROS analysis by flow cytometry

After treatments for 24 h, the PANC-1 cells were collected and washed twice with PBS. The cells were then resuspended in PBS and incubated with 5 μM dihydroethidium (DHE) (Sigma-Aldrich) for 30 min at 37°C in the dark. The fluorescence intensity was immediately measured using flow cytometry. Statistical analysis of fluorescence was recorded by FACSCanto™ II system (BD Biosciences, San Jose, CA, USA).

### Monodansylcadaverine (MDC) staining

The PANC-1 cells were grown on sterile glass coverslips and treated with different conditions for 24 hours. After being washed twice with PBS, the cells were incubated with 50 μM MDC (Sigma-Aldrich) for 30 min at 37°C in dark. Subsequently, the coverslips were carefully washed and mounted with 50% glycerol in PBS. The observation of MDC-stained vesicles was conducted using Zeiss Axio Imager A1 fluorescence microscope.

### Western blotting analysis

After treatments for 24 h, the PANC-1 and HepG2 cells were collected and briefly washed with PBS. Then, the cells were lysed in lysis buffer (50 mM Tris-HCl, pH 7.4, 0.15 M NaCl, 0.25% deoxycholic acid, 1% NP-40, 1% Triton X-100, 0.1% SDS, 1 mM EDTA) with fresh protease and phosphatase inhibitor cocktail (Millipore, Billerica, MA, USA) on ice for 30 min. The protein concentrations were determined by BSA method after the centrifugation of cell lysates. Equal amounts of protein extract were separated by 12% SDS-PAGE and electroblotted onto polyvinylidene difluoride (PVDF) membranes (Millipore). The membranes were blocked for 1 h with 50 g/l skim milk in TBST washing buffer (20 mM Tris, 150 mM NaCl, and 0.1% Tween 20) and then incubated overnight with primary antibodies at 4°C, followed by incubation with designated secondary antibodies for 1 h. In this study, the employed primary antibodies were illustrated as following: anti-β-actin, anti-LC3, anti-PARP, and anti-cleaved PARP (Gentex, Irvine, CA, USA); anti-phospho-Akt (Ser473), anti-cleaved caspase-3, and anti-cleaved caspase-9 (Cell Signaling Technology, Danvers, MA, USA). The secondary antibodies were obtained from Jackson ImmunoResearch Laboratories (West Grove, PA, USA). According to the manufacturer's instructions, each antibody was diluted to its optimal concentration. Subsequently, the proteins were visualized using chemiluminescence ECL kit (T-Pro Biotechnologies, New Taipei City, Taiwan). β-actin was used as the internal control to correct relative levels of each protein loading.

### Statistical analysis

Each experiment was carried out three times for validation, and statistical analysis was performed using SigmaPlot version 12.5 for Windows (Systat Software, Inc., San Jose, CA, USA). All results are expressed as the mean percentages of dead cells of three independent experiments ± the standard error of the mean. The one-way analysis of variance (ANOVA) was employed for comparing two treatment groups, and a value of P < 0.05 was considered statistically significant. In the figures, * is used for P < 0.05, ** for P < 0.01, and *** for P < 0.001.

### Synergy quotient calculation for synergism

For the combination of a phytochemical and two types of physical stimulation, the synergism quotient (SQ) was evaluated by calculating the growth inhibition rate of each treatment and then dividing the net inhibition rate of the combination treatment [A + B + C] by the sum of individual inhibition rates [A] + [B] + [C]. SQ greater than 1.0 reveals a synergistic effect [28].

## Results

### Application of the low strength PEF and low energy US enhanced the inhibition effect of EGCG on PANC-1 cells

**Fig 2:**
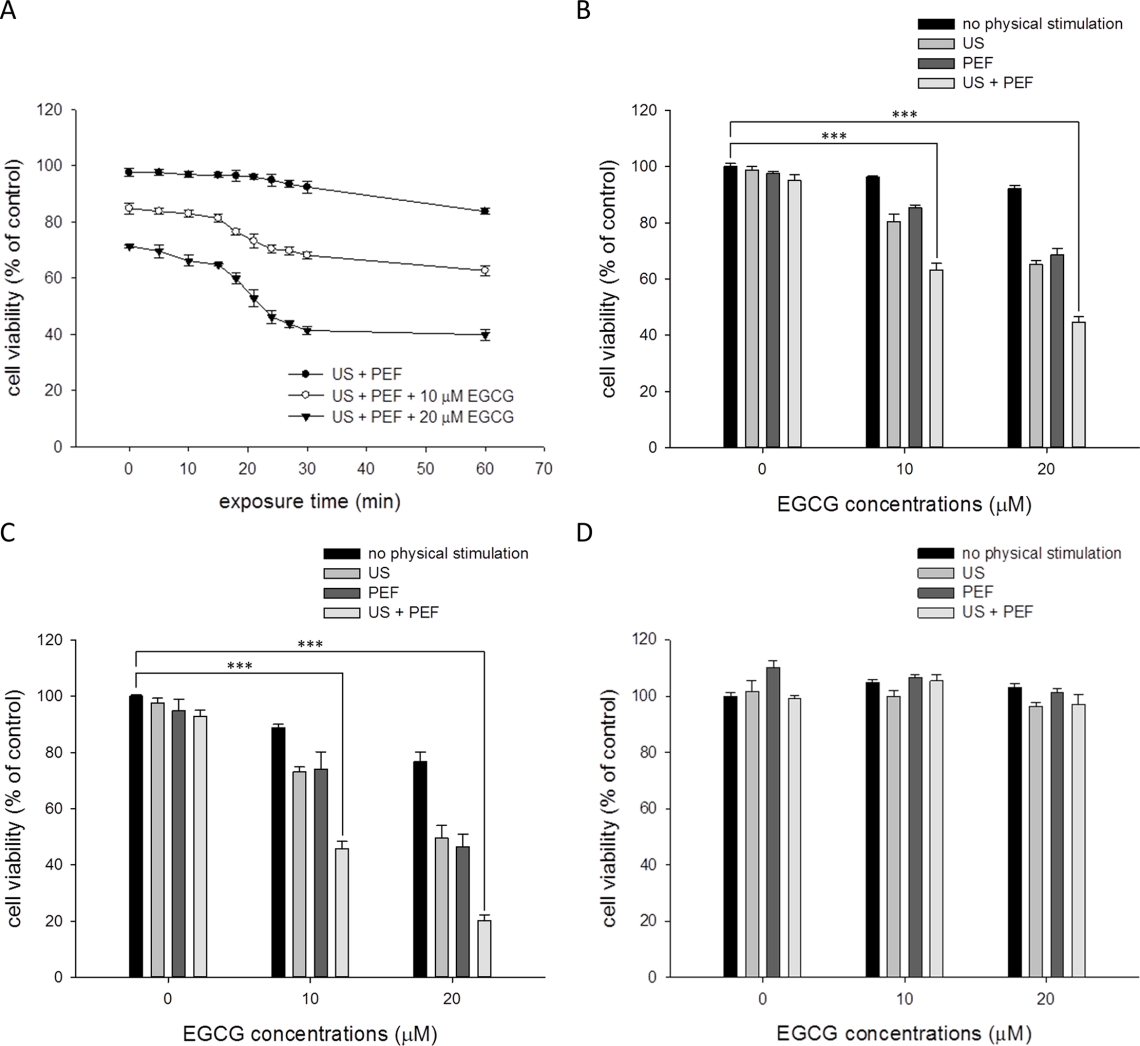
Effects of the combined triple treatment on the viability of the PANC-1 cells. After treatment for 24 h and 72 h, the cell viability was determined using MTT assay. (A) The PANC-1 cells were treated with different duration of 0.3 W/cm^2^ US exposure (0 to 60 min) alone or combined with the EGCG (10 or 20 μM) and consecutive PEF (60 V/cm in amplitude, 2 ms in duration, and 2 Hz in frequency) for 24 h. Single or combined treatments of the EGCG (10 or 20 μM), 60 V/cm PEF, and 30 min US were applied to the PANC-1 cells for 24 h (B) or 72 h (C). (D) Single or combined treatments of the EGCG (10 or 20 μM), 60 V/cm PEF, and 30 min US were applied to the HEK293 cells for 24 h. (*** is used for P < 0.001).

To investigate whether the low strength PEF and low energy US could enhance the ability of EGCG to inhibit the pancreatic carcinoma, we first study the effect of the PEF and US on the cell viability of PANC-1 cells treated with the EGCG. As shown in Fig 2A, based on our previous study [4] and numerical investigations about intracellular temperature distribution induced by US [25, 26], the 0.3 W/ cm^2^ US treatments with various exposure time (0 to 60 min) were applied to the PANC-1 cells co-treated with 60 V/cm PEF and EGCG (0 to 20 μM). After the treatments, the growth inhibition effects for the combined treatments were evaluated using MTT and ATP assays. The result showed that the double treatment of the US and PEF had little effect on the cell viability of PANC-1 even when the cells were exposed to the US for 60 min. On the contrary, when the cells were triple treated with the US, PEF, and EGCG (10 or 20 μM), the cell viability was dramatically decreased once the US exposure time was longer than 15 min. We then compared the inhibition effects between each single treatment and the triple treatment to better understand the benefit of this combined triple treatment. As shown in Fig 2B and S1 Fig, the PANC-1 cells were treated with EGCG (0 to 20 μM) alone or combined with various physical stimulations for 24 h. The result showed that the viability inhibition effect of the double treatment of the EGCG and PEF was similar with that of the double treatment of the EGCG and US, and the triple treatment exhibited the optimal inhibition effect on the PANC-1 cells. When the cells were treated with the triple treatment for 72 h, as shown in Fig 2C and S2 Fig, the cell viability was reduced almost to 20% of the control by SQ greater than 2.5. This revealed that the *in vitro* anticancer efficacy of the non-invasive combined triple treatment would be close to that of the chemotherapy drugs when the triple treatment is characterized as a long-term treatment. Following, the human embryonic kidney cells HEK293 were used as a non-cancerous model to investigate whether the inhibition effects of the triple treatment were selective in dealing with cancer cells. As shown in Fig 2D and S3 Fig, the result revealed that the HEK293 cells were not affected by each single treatment. Furthermore, the cell viability of the HEK293 cells was not obviously reduced after the triple combined treatment. Collectively, these results proposed that the application of the PEF and US could profoundly improve the EGCG-induced inhibition effects on the cancer cells, and this triple treatment was specific for PANC-1 cells. Based on these results, the following experiments were carried out using 20 μM EGCG, 60 V/cm PEF, and 30 min US exposure.

### The exposure of PANC-1 cells to the low energy US increased intracellular ROS generation and acidic vesicles

**Fig 3:**
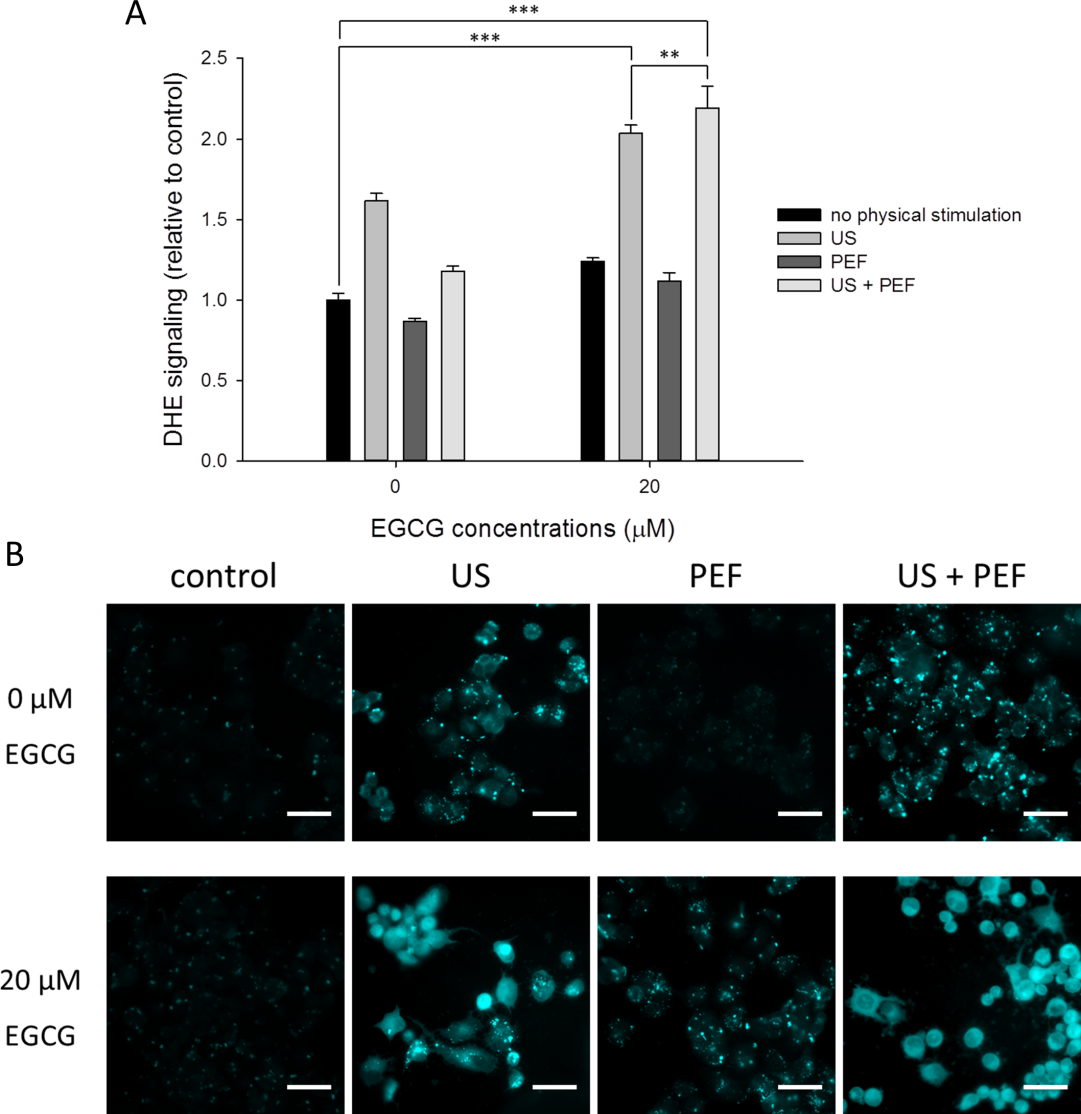
Effects of the combined triple treatment on the ROS level and acidic vesicles in the PANC-1 cells. (A) Dihydroethidium (DHE) was used to evaluate the level of superoxide radical anion (O_2_^•−^) in the PANC-1 cells. The result showed that the US exposure could increase the ROS level, and the triple treatment demonstrated the highest accumulation of ROS. (B) MDC staining was employed to observe the acidic vesicles in the PANC-1 cells. The result showed the MDC-labeled vesicles were significantly increased in the cells with triple treatment when compared with the untreated control. Scale bar = 20 μm. (** is used for P < 0.01 and *** for P < 0.001).

We further investigated whether the reactive oxygen species (ROS) were increased in response to the triple treatment, so the dihydroethidium (DHE) fluorescence was measured to analyze the level of superoxide radical anion (O_2_^•−^). As shown in Fig 3A, the DHE fluorescence intensities were faintly increased when the cells were single or double treated with 20 μM EGCG and 60 V/cm PEF. In contrast, as long as the 30 min US exposure was applied to the PANC-1 cells, the level of cytosolic ROS was notably increased. Besides, the increment was significantly promoted when the cells were double treated with the US and EGCG, and the triple treatment demonstrated the highest accumulation of ROS.

Since it has been shown that superoxide radical anion (O_2_^•−^) is the major ROS for regulating autophagy [29], we further employed MDC staining to investigate whether the accumulation of the ROS would induce autophagy. It was found that the number of MDC-labeled vesicles was significantly increased in the cells double treated with the US and EGCG (Fig 3B). In addition, a comparable staining pattern was observed in the triple treatment. Since the levels of the MDC staining followed a similar trend to those of the ROS, we speculated that the application of the US in the triple treatment would initiate the autophagy pathway to affect cell death through the increment of ROS.

### Double treatment of the low energy US and EGCG enhanced the autophagy signaling

**Fig 4:**
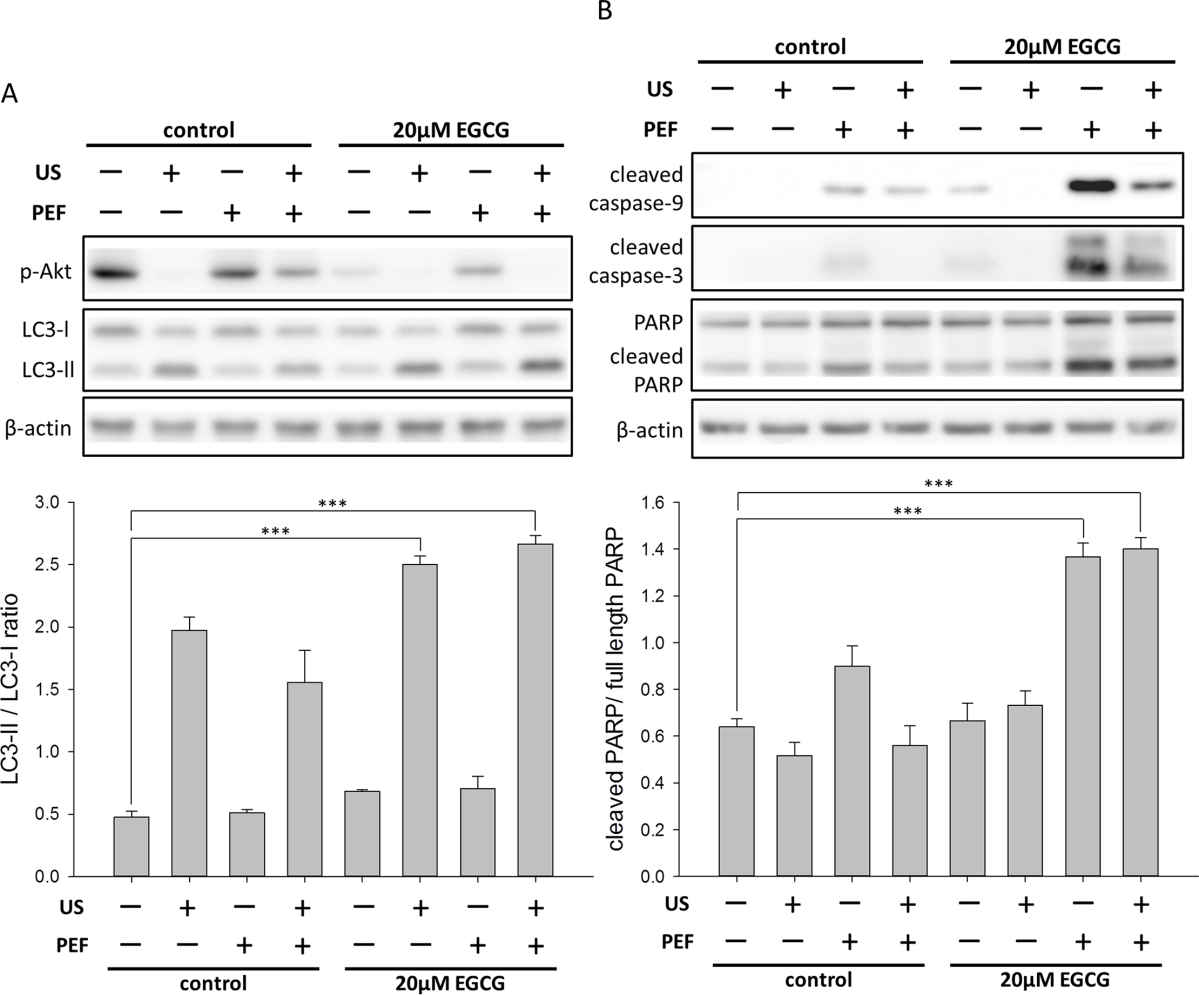
Effects of the combined triple treatment on the intracellular proteins in the PANC-1 cells. The protein expression levels of p-Akt, LC3-II, PARP, cleaved caspase-9, and cleaved caspase-3 were measured using Western blot analysis. β-actin was used as a loading control. (A) The triple treatment decreased the phosphorylation of Akt protein and increased the conversion of LC3-I to LC3-II. The band intensities of LC3-II and LC3-I were quantified to calculate the LC3-II/LC3-I ratio. (B) Apoptotic proteins were activated after the cells were treated with the combined triple treatment. The cleaved PARP was quantified relative to the full-length PARP. These results suggested that both the apoptosis and the autophagy were triggered in the PANC-1 cells by the triple combined treatment. (*** is used for P < 0.001).

The most commonly used approach to evaluate autophagy is to measure the switch of LC3-I to LC3-II, which is covalently attached to the phagophore membrane in the process of autophagosome formation [30]. As shown in Fig 4A, the level of LC3-II was obviously increased in the cells double treated with 30 min US and 20 μM EGCG, and a similar result was obtained when the cells were treated with the triple treatment. Because the level of LC3-II was also increased in the cells treated with the US alone, it seems that the single application of US exposure to the PANC-1 cells could induce the initiation of autophagy. It has been reported that the PI3K/Akt/mTOR signaling pathway is involved in the anticancer effect of EGCG [31], and this pathway is also the upstream signaling pathway to regulate autophagy [32]. We thus studied the activated p-Akt to understand the mechanism underlying the triple treatment-induced inhibition effect. As shown in Fig 4A, the level of p-Akt was obviously affected by a single treatment of the US. We also found the phosphorylation of Akt was reduced in the cells treated with 20 μM EGCG alone. In addition, the reduction was dramatically enhanced by the double treatment of the EGCG and US, and a similar result was obtained in the triple treatment. Taken together, these results suggested that the triple treatment may induce a cytotoxic autophagy through the down-regulation of p-Akt and the up-regulation of LC3-II.

### Double treatment of the low strength PEF and EGCG induced caspase activities

In comparison with the double treatment of 30 min US and 20 μM EGCG, we next studied whether the PEF in the triple treatment could still induce the apoptosis as we previously reported [4]. It is known that the activation of caspases plays an important role in the signal transduction of apoptosis, so we quantified the endogenous levels of cleaved caspase-9 and -3 in the PANC-1 cells treated with the triple treatment. As shown in Fig 4B, when compared with the double treatment of the EGCG and US, the level of cleaved caspase-9 was significantly increased in the cells with the double treatment of the PEF and EGCG. When the cells were treated with the combined triple treatment, the level of cleaved caspase-9 was slightly reduced. Accordingly, similar expression levels were obtained in the cleaved caspase-3 observation. It was also observed that the combined triple treatment promoted the expression of cleaved PARP, which is an indicator of the cells undergoing apoptosis. These data revealed that, in the triple treatment, the PEF stimulation subsequent to the EGCG and US treatment initiated the activation of caspase signaling and induced apoptosis to further enhance the inhibitory effects on PANC-1 cells. It is noteworthy that the double treatment of the PEF and EGCG induced the highest level of caspase activity (Fig 4B); however, as shown in Fig 2B and 2C, the growth inhibitory effect of this double treatment on PANC-1 cells was still lower in comparison with that of the triple treatment. Consequently, these results were consistent with the previous suggestion that the triple treatment may induce a cytotoxic autophagy rather than a cytoprotective [33], so the triple treatment-induced apoptosis and autophagy did not counteract each other but cooperatively enlarged the inhibition effects.

### Both autophagy and apoptosis inhibitors attenuated the triple treatment-induced inhibition effects

**Fig 5:**
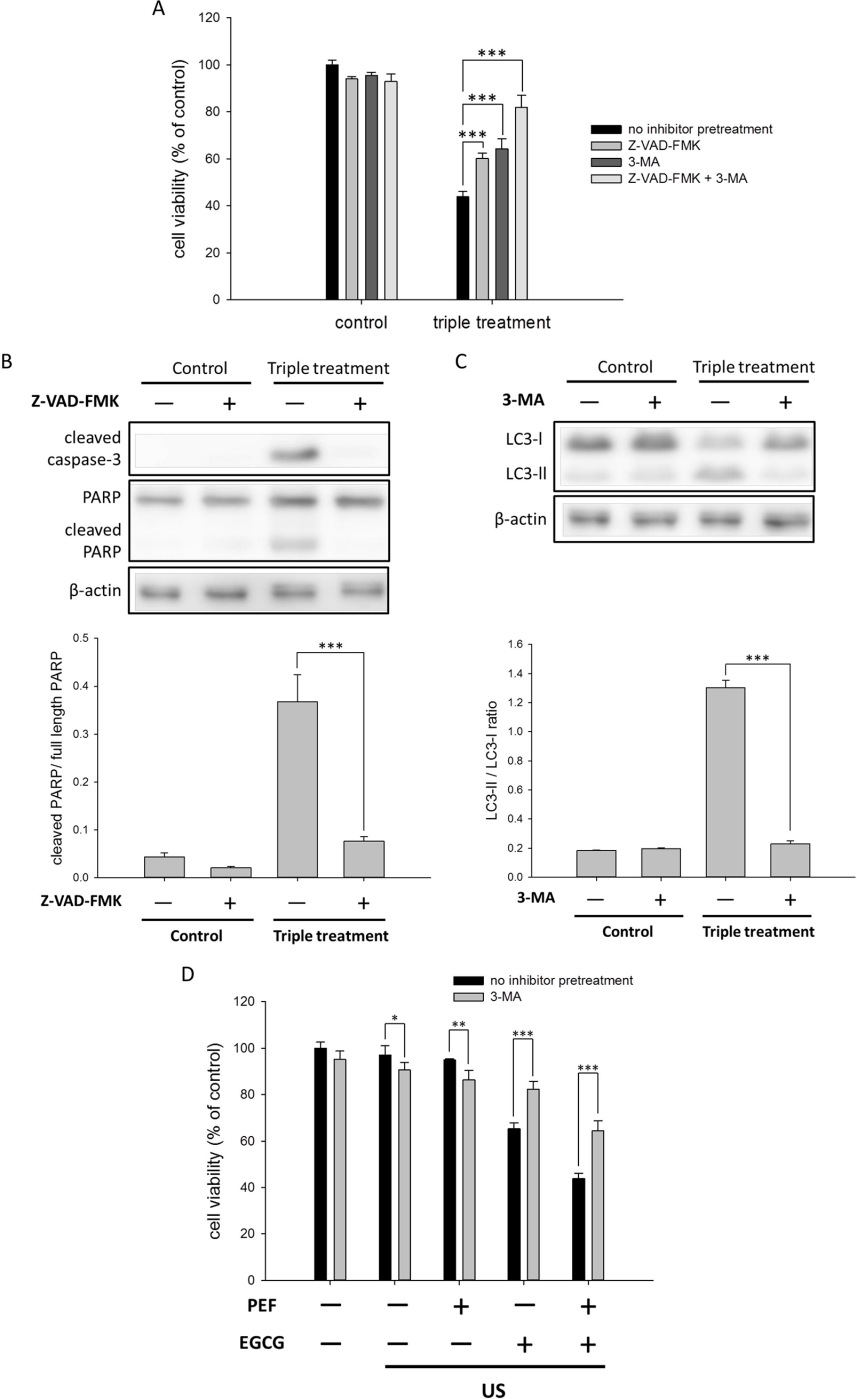
Effects of the autophagy and apoptosis inhibitors on the triple treatment-induced growth inhibition in the PANC-1 cells. The 3-MA and Z-VAD-FMK were used for the autophagy and apoptosis inhibitor analysis, respectively. (A) The PANC-1 cells were pretreated with the autophagy inhibitor 3-MA and the caspase inhibitor Z-VAD-FMK before the triple treatment for 24 h. The result showed that both 3-MA and Z-VAD-FMK could partially enervate the triple treatment-induced growth inhibition in the PANC-1 cells. (B) The pretreatment of Z-VAD-FMK significantly reduced the levels of cleaved caspase-3 and cleaved PARP. The cleaved PARP was quantified relative to the full-length PARP. (C) The pretreatment of 3-MA blocked the conversion of LC3-I to LC3-II. The band intensities of LC3-II and LC3-I were quantified to calculate the LC3-II/LC3-I ratio. β-actin was used as a loading control for each Western blot assay. (D) To better understand the role of autophagy in the triple treatment, 3-MA pretreatment was applied to the combination treatments using US stimulation. (* is used for P < 0.05, ** for P < 0.01, and *** for P < 0.001).

To further confirm that the inhibition effects of the triple treatment were induced by both the autophagy and apoptosis, we pretreated the cells with the autophagy inhibitor 3-MA and the caspase inhibitor Z-VAD-FMK before the triple treatment. As shown in Fig 5A, the viability of the control cells was only slightly affected by the single or the double application of Z-VAD-FMK and 3-MA. The cells were moderately rescued when Z-VAD-FMK was applied to the triple treatment, and a comparable restoration was observed when 3-MA was applied to the triple treatment. Moreover, the double application of 3-MA and Z-VAD-FMK significantly recovered the triple treatment-induced reduction in the cell viability of the PANC-1 cells. The endogenous levels of autophagy- and apoptosis-related proteins were also evaluated when 3-MA and Z-VAD-FMK were separately applied to the triple treatment. As shown in Fig 5B, the results of the Western blotting assay revealed that the pretreatment of Z-VAD-FMK significantly reduced the level of cleaved caspase-3, which was activated in the triple treatment. Consistent with the observation of the cleaved caspase-3, the cleaved PARP was also reduced, suggesting that the blocking of the caspase activities attenuated the inhibition effects of the triple treatment. In addition, the pretreatment of the cells with 3-MA before the triple treatment significantly reduced the conversion of LC3-I to LC3-II (Fig 5C). This data implied that the application of 3-MA to the triple treatment suppressed the LC3-II activation and blocked the formation of the autophagosome, resulting in the restoration of the cell viability. As shown in Fig 5D, the applications of 3-MA to both of the single US treatment and the double treatment of the US and PEF were found to slightly decrease the viability of PANC-1 cells; however, the applications of 3-MA to both of the triple treatment and the double treatment of the EGCG and US were observed to obviously increase the cell viability. This indicated that the US-triggered autophagy was switched from the cytoprotective to the cytotoxic when the US stimulation was combined with the EGCG treatment. Collectively, our results proposed that the combination of the US and EGCG induced cytotoxic autophagy, and the incorporation of the PEF and EGCG synergistically triggered the caspase-dependent apoptosis to elevate the inhibition effects. Moreover, according to the results of the triple treatment applied with the inhibitors, it seems that the triple treatment-induced autophagy and apoptosis may independently contribute to cell death. Notably, the double application of Z-VAD-FMK and 3-MA in the triple treatment did not attenuate the cell death completely. This result revealed that other cell death mechanisms beyond the apoptosis and autophagy pathways may also be involved, and the detailed mechanism of cell death deserves further investigation.

### The triple treatment overcame the cytotoxicity tolerance of HepG2 cell to EGCG and significantly reduced its cell viability

**Fig 6:**
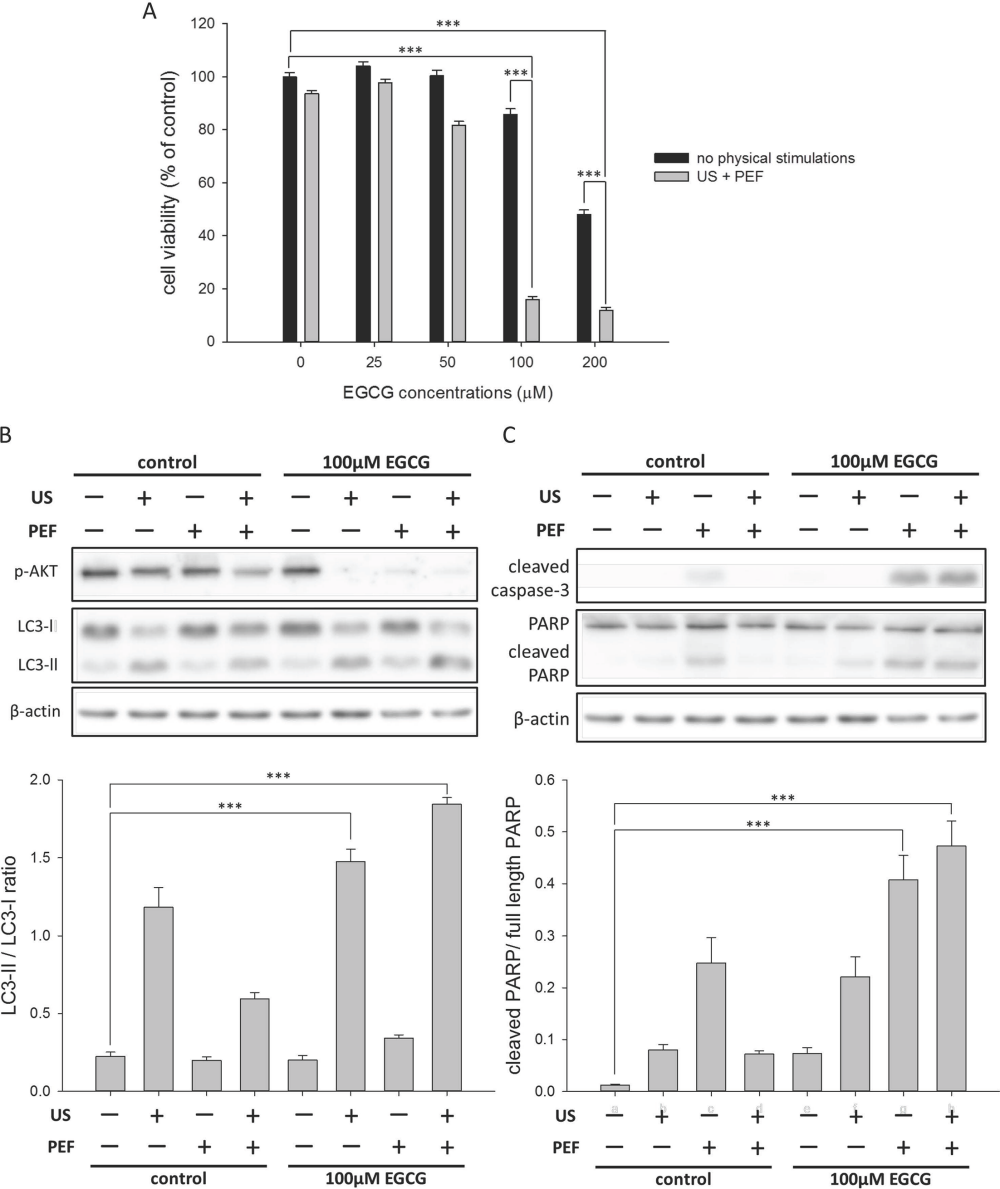
Effects of the combined triple treatment on the viability of the HepG2 cells. The cell viability (24 h) was determined using MTT assay. (A) The HepG2 cells were triple treated with the EGCG (0 to 200 μM), 0.3 W/cm^2^ US exposure (60 min), and consecutive 60 V/cm PEF for 24 h. The result revealed that the triple treatment could overcome the tolerance of cancer cells to EGCG and cause the death of HepG2 cells. (B) The triple treatment decreased the phosphorylation of Akt protein and increased the conversion of LC3-I to LC3-II in the HepG2 cells. The band intensities of LC3-II and LC3-I were quantified to calculate the LC3-II/LC3-I ratio. (C) The triple treatment triggered the activations of caspase-3 and PARP in the HepG2 cells. The cleaved PARP was quantified relative to the full-length PARP. β-actin was used as a loading control for each Western blot assay. (*** is used for P < 0.001).

Following, we applied the combined triple treatment to the HepG2 cells to examine the inhibition efficacy on the hepatic cancer cells. We found the cell viability was still high even when the cells were single treated with 100 μM EGCG (Fig 6A and S4 Fig). This revealed that HepG2 cells were characteristic of high tolerance to EGCG-induced cytotoxicity, which was similar to that observed in the same HepG2 system employed with doxorubicin and daunorubicin [34]. However, the viability of HepG2 cells was drastically decreased after both 60 V/cm PEF and 60 min US were co-utilized in the treatment of 100 μM EGCG. This result revealed that the triple treatment could overcome the tolerance of cancer cells to EGCG and significantly enhance the inhibition effects of EGCG to cause the death of HepG2 cells. In addition, considering that the employed US exposure time and the concentration of EGCG were different between the remedy for the PANC-1 and for the HepG2 cells, it suggested that the optimal parameters of the triple treatment were cell line-dependent. We further analyzed the effects of the triple treatment on the intracellular signaling proteins in the HepG2 cells. As shown in Fig 6B, the level of p-Akt was significantly decreased, and the amount of LC3-II was obviously increased in the cells with the combined triple treatment, in consistent with the observations in the PANC-1 cells. Additionally, it was also found that the triple treatment triggered the activations of caspase-3 and PARP in the HepG2 cells (Fig 6C). Therefore, these results suggested that both the autophagy and apoptosis were induced in the same way in the HepG2 cell, and the two pathways synergistically caused the death of this tenacious cell.

## Discussion

Variations of combination therapy have been developed against malignant tumors for over 50 years [35], and the combination of different anticancer methods to target multiple pathways [36] is believed to be more efficient and has gained popularity [2, 37, 38]. With respect to other diseases, combination therapies have been performed with clinical success, such as the triple cocktail for the HIV infection and the triple therapy for the treatment of Helicobacter pylori. Here, we have demonstrated a novel treatment combining low strength PEF, low energy US, and EGCG to combat the cancer. To the best of our knowledge, this is the first study that has provided a non-invasive anticancer treatment employing one herbal administration and two types of physical stimulation with moderate parameters. Our results showed that the EGCG-induced inhibition effects were significantly enhanced by the double application of the US exposure and the PEF stimulation, and the viability of the cancer cells was reduced to merely 20% of the control, suggesting this combined triple treatment had a similar *in vitro* efficacy as the chemotherapy drugs.

An advantage of the triple treatment is lowering the concentration of EGCG for treatment. In this study, the application of the US exposure and the PEF stimulation to the EGCG treatment significantly reduced the viability of PANC-1 and HepG2 cells, when compared with the single EGCG treatment. By means of the physical forces, the triple treatment was capable of overcoming the high tolerance of HepG2 cells to the EGCG-induced cytotoxicity and improving the inhibition efficiency of EGCG. Besides, a previous study had shown that EGCG could be loaded in the colloidal carriers, which are conjugated with cancer-specific peptides [39]. Furthermore, it has been reported that the US can locally trigger the release of anticancer agents from the liposomes [40]. Therefore, adopting these technologies to release the EGCG precisely in the target cell and to reduce the metabolic rate of EGCG in the body, this combined triple treatment could even induce the oxidation of EGCG by the US stimulation, and hence, would provide a most promising anticancer method against various cancer cells.

The application of US to the anticancer treatments is appealing, because the non-invasive US can penetrate deeper into the tissues and can be flexibly focused at a specific region. In this study, low energy US was employed in the triple treatment, and this combined treatment synergistically triggered the activation of the cytotoxic autophagy. We found that the exposure of US increased the levels of ROS in the PANC-1 cells. This is consistent with previous studies which reported that oxidative stress is the key mechanism involved in the US-induced bio-effects [41–43]. In addition, some studies have shown that the US-induced accumulation of ROS can be attributed to the collapse of cavitation bubbles [44, 45], which occurs when a liquid undergoes rapid changes of pressure. In our study, we observed that the triple treatment significantly generated superoxide radical anions (O_2_^•−^) when compared with each the single treatment. Since the oxidation reactions of EGCG result in the generation of ROS species [6, 46], we proposed that the application of the US in the triple treatment not only induced the cavitation bubbles, but also increased the molecular energy and collision frequency, and therefore the superoxide radical anions (O_2_^•−^) were excessively produced. Our further results of the MDC staining and the Western blot assay provided the evidence that the overproduction of superoxide radical anion (O_2_^•−^) may play an important role in the activation of autophagy. Moreover, we also found that the application of the US dramatically intensified the EGCG-induced inhibition of Akt phosphorylation. It is important to note that the single treatment of the US increased the activation of LC3-II and also induced autophagosomes (see Fig 3B), but the cell viability shown in Fig 2B and 2C, respectively, were only slightly affected. In contrast, the triple treatment which triggered the autophagy pathway as well was found to significantly reduce the cell viability (24 h and 72 h) of cancer cells (see Fig 2B and 2C). Consequently, our results suggest that the drastically inhibited phosphorylation of Akt and the overaccumulation of ROS could be attributed to the cooperative effects of the EGCG and US stimulation, and hence, the US-triggered autophagy was switched from the cytoprotective to the cytotoxic in the combined triple treatment, evidenced by the 3-MA experiments.

The lipid bilayer is an essential structure constituting the intracellular vesicles, which provide isolated compartments and participate in the cell metabolism as well as the signaling transduction. As amply reported in previous studies, the autophagy pathway is initiated through the nucleation and the elongation of an isolated membrane, followed by the fusion with a lysosome to form an autolysosome where sequestered materials are degraded [47]. It has been further demonstrated that the bilayer membranes would execute an oscillation motion in response to the acoustic pressure induced by the US, and the two leaflets of the lipid bilayer might be separated and bent [48]. In addition, a previous study showed that EGCG could alter the lipid organization and reduce the bilayer stiffness [49]. Thus, the significantly facilitated formation of autophagy in the triple treatment could be partly attributed to the decreased stiffness of lipid membrane caused by the EGCG and the forced oscillation induced by the US. Moreover, the inhibitor experiments presented here have revealed that the triple treatment-induced autophagy was cytotoxic and led to cell death. Collectively, the triple combined treatment synergistically increased the generation of ROS, enhanced the formation of autophagosome, and suppressed the phosphorylation of Akt to reduce cell survival. As a result, this remedy has promoted the cytotoxic autophagy and efficiently inhibited the cancer cells.

In point of fact, there are different forms of programmed cell death reported in recent studies, such as apoptosis, autophagy, and programmed necrosis [36]. Recently, we have reported a combination treatment employing the EGCG and the low strength PEF to combat against PANC-1 cells [4]. We observed that the low strength PEF could enhance the anticancer ability of EGCG due to the synergistic disturbance of the mitochondrial membrane potential (MMP). Besides, the migration ability of the PANC-1 cells was significantly suppressed when the cells were co-treated with the PEF and EGCG. In this present work, we found that the triple treatment can still increase the caspase activity, and the triggered apoptosis contributed to the triple treatment-induced inhibition effects, evidenced by the rescue of the cancer cells pretreated with Z-VAD-FMK in the triple treatment. We speculated that the mechanism underlying the synergistic effects induced by the PEF might be similar to our previous results, which demonstrated a significant dissipation of the MMP and the enhanced mitochondria-dependent apoptosis when the cells were co-treated with the EGCG and PEF. Since the autophagy induced by the triple treatment was switched from cytoprotective to cytotoxic, the PEF-enhanced apoptosis was no longer counteracted by the autophagy but cooperatively led to the significant inhibition of the cell viability. In this study, we further investigated the therapeutic efficacy of this triple treatment in the hepatoma cell line HepG2. We obtained a similar result that the autophagy and apoptosis were simultaneously activated and concurrently caused the significant cell death of the HepG2. Notably, by means of adopting different remedy parameters than that applied to the PANC-1 cells, the triple treatment could overcome the high tolerance of HepG2 cells to the EGCG-induced cytotoxicity and make the cancer cells more sensitive to the EGCG treatment. Therefore, we speculate that our triple treatment with different parameters should exhibit a similar excellent efficacy when it is employed to deal with various types of cancer.

Last but not least, we have observed that the EGCG stock solution treated with sonication dispersion showed a similar and slightly promoted anticancer effect in comparison to the EGCG stock solution with no treatment (S5 Fig). This indicates that the triple treatment-induced anticancer effects may also be attributed to the indirect inhibitory effects of EGCG, and the results are also consistent with the previous studies that have reported the oxidative products of EGCG can exhibit significant cytotoxic effects [6, 7]. On the other hand, it has been reported that the auto-oxidation of EGCG in a cell culture system [5] may not occur in internal organs due to the fact that the oxygen partial pressure in the tissues is much lower than that in the cell culture condition [50]. Nevertheless, we suggest that the US stimulation in the triple treatment can generate superoxide radical anions (O_2_^•−^) through the cavitation reactions [51] and thus may cause the oxidation of EGCG, triggering the oxidation-mediated anticancer effects *in vivo*. Therefore, we speculated that the EGCG oxidation-dependent and EGCG oxidation-independent pathways would simultaneously contribute to the triple treatment-induced cell death in internal organs, and the clear distinction between these two pathways should be carried out in future studies to gain a better understanding about the roles of EGCG and EAOPs in the combined triple treatment.

In conclusion, we have provided the first demonstration that the US and PEF were applied together to the EGCG treatment to inhibit cancer cells, and the results of this study have revealed that the inhibition ability of EGCG was dramatically enhanced by the further application of these two physical stimulations. Since both the autophagy and apoptosis were simultaneously activated to cause cell death, our triple treatment showed a significant potential of the combination treatment with the non-invasive physical stimulations and a herbal administration in the anticancer treatment. After all, the development of an anticancer treatment with a beneficial efficiency, less suffering, fewer side effects, and low medical expenses is an important benefit for patients. Here, our study gives an excellent model in that the natural products applied with mild physical stimulations can induce multiple cell death pathways and drastically inhibit the viability of cancer cells. Therefore, further investigations for optimization and therapeutic application of this treatment should be launched forthwith.

## Supporting information

S1 Fig Effects of the combined triple treatment on the PANC-1 cell viability. After treatment for 24 h, the viability of PANC-1 cells was measured using ATP content-based method. (*** is used for P < 0.001).
(TIF)

S2 Fig Effects of the combined triple treatment on the PANC-1 cell viability. After treatment for 72 h, the viability of PANC-1 cells was measured using ATP content-based method. (*** is used for P < 0.001).
(TIF)

S3 Fig Effects of the combined triple treatment on the HEK293 cell viability. After treatment for 24 h, the viability of HEK293 cells was measured using ATP content-based method. (*** is used for P < 0.001).
(TIF)

S4 Fig Effects of the combined triple treatment on the HepG2 cell viability. After treatment for 24 h, the viability of HepG2 cells was measured using ATP content-based method. (*** is used for P < 0.001).
(TIF)

S5 Fig Effects of the sonication dispersion of EGCG solution on the triple treatment-induced anticancer effects. EGCG stock solution was treated with or without the sonication dispersion, and then the solutions were used for the combined triple treatment. After treatment for 24 h or 72 h, the viability of PANC-1 cells was measured using MTT assay.
(TIF)

S1 File Raw data of MTT assay. (RAR)

S2 File Raw data of ATP-based viability assay. (RAR)

S3 File Raw data of DHE flow cytometry. (RAR)

S4 File Raw image of MDC staining. (RAR)

S5 File Raw images of PANC-1 proteins. (RAR)

S6 File Raw data of inhibitors. (RAR)

S7 File Raw data of HepG2 proteins. (RAR)

S8 File Raw data of EGCG sonication. (RAR)
