# Supplementary figures and images for: Studies on the non-invasive anticancer remedy of the triple combination of epigallocatechin gallate, pulsed electric field, and ultrasound
Source: PLoS One. 2018 Aug 6;13(8):e0201920. doi: 10.1371/journal.pone.0201920 (PMC6078317; doi:10.1371/journal.pone.0201920)

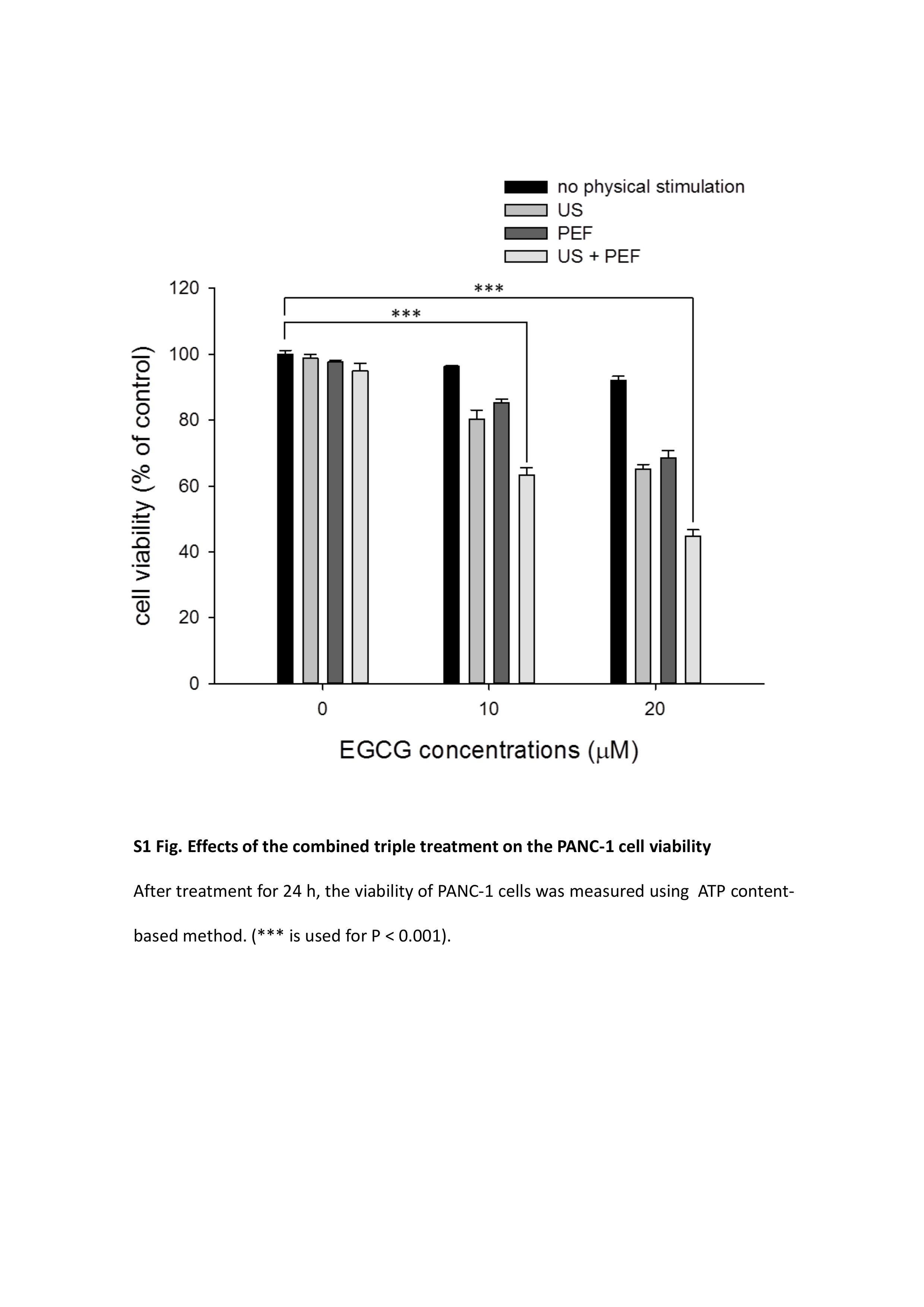

Supplement: S1 Fig — After treatment for 24 h, the viability of PANC-1 cells was measured using ATP content-based method. (*** is used for P < 0.001). (TIF) [file pone.0201920.s001.tif]

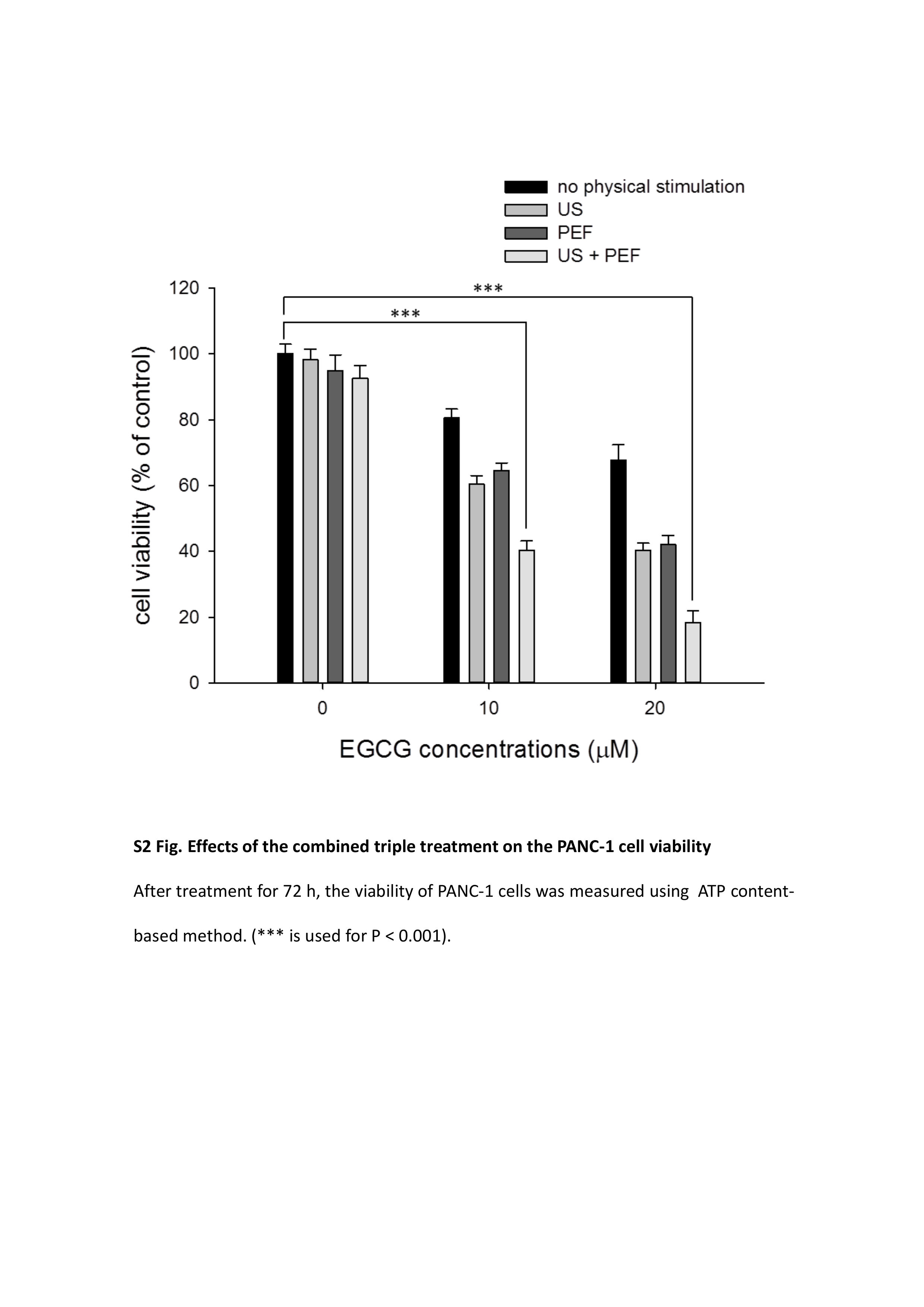

Supplement: S2 Fig — After treatment for 72 h, the viability of PANC-1 cells was measured using ATP content-based method. (*** is used for P < 0.001). (TIF) [file pone.0201920.s002.tif]

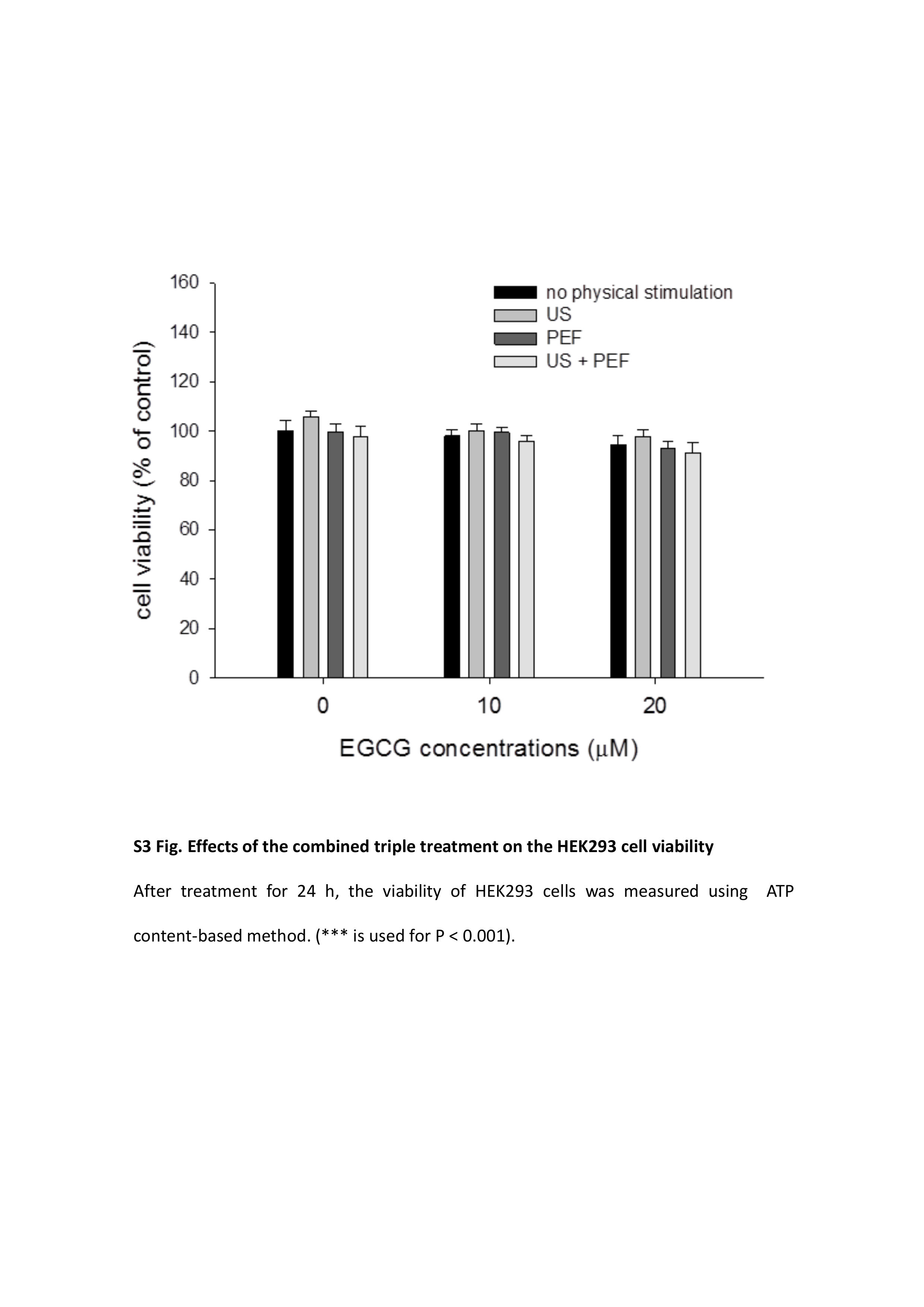

Supplement: S3 Fig — After treatment for 24 h, the viability of HEK293 cells was measured using ATP content-based method. (*** is used for P < 0.001). (TIF) [file pone.0201920.s003.tif]

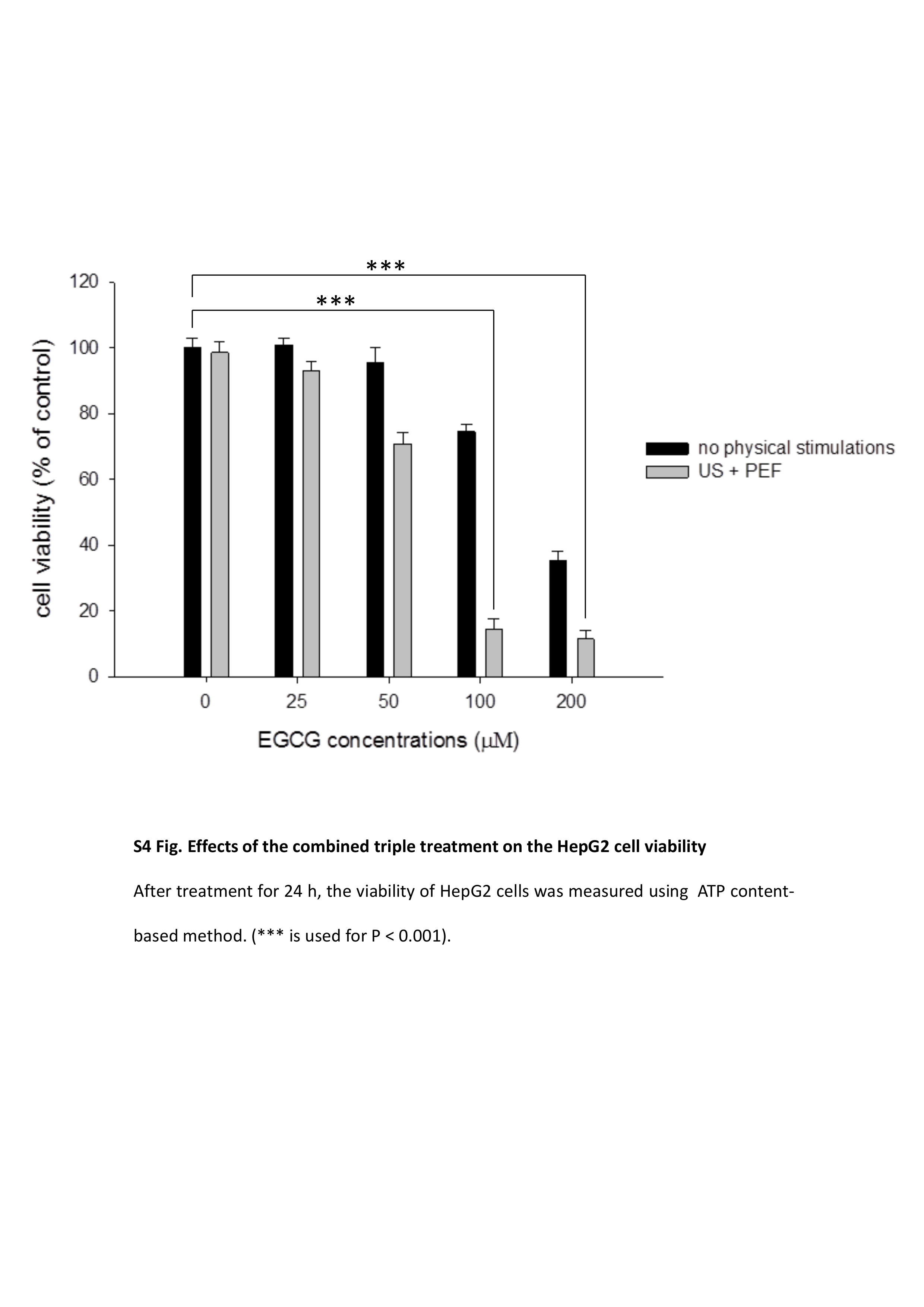

Supplement: S4 Fig — After treatment for 24 h, the viability of HepG2 cells was measured using ATP content-based method. (*** is used for P < 0.001). (TIF) [file pone.0201920.s004.tif]

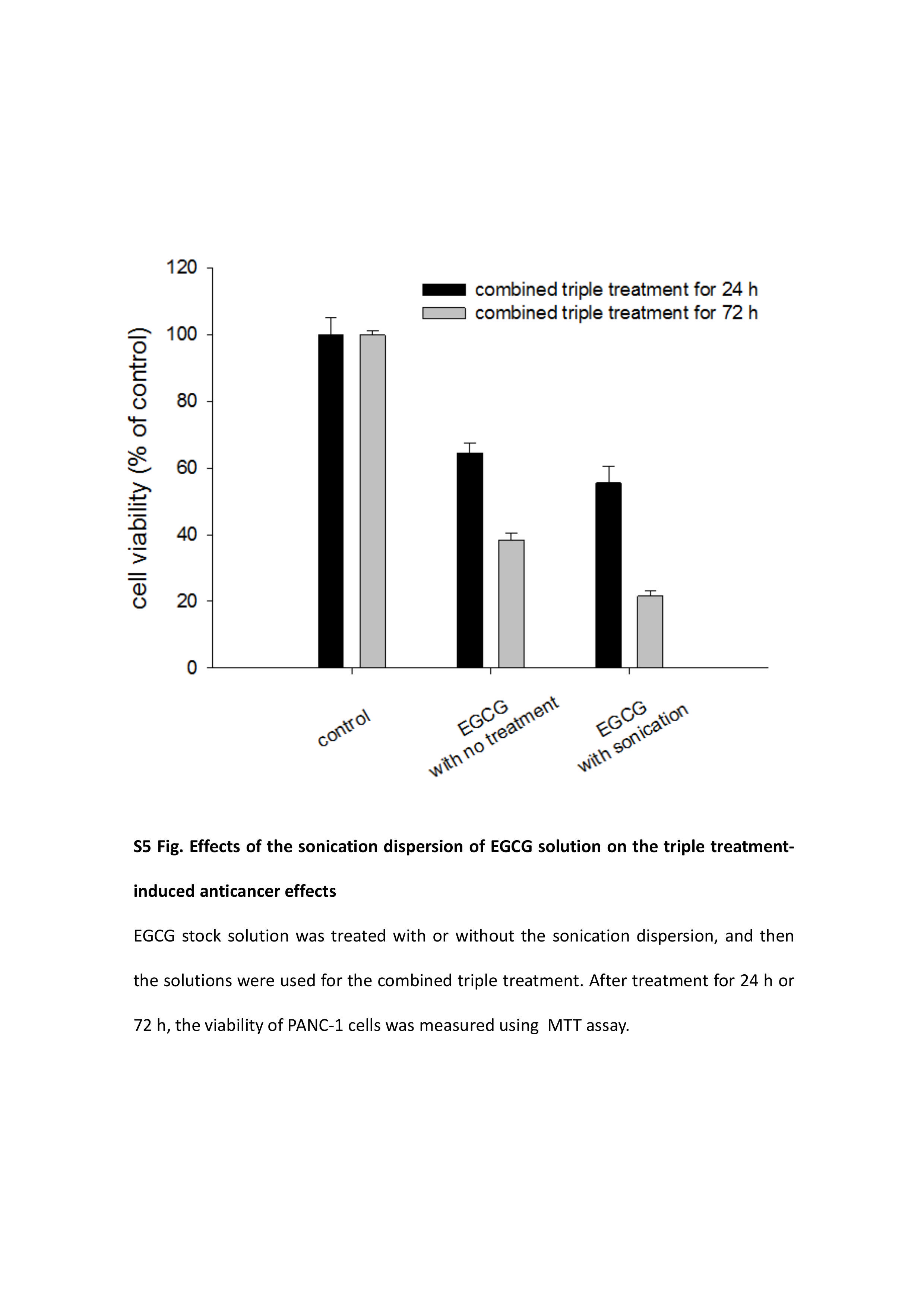

Supplement: S5 Fig — EGCG stock solution was treated with or without the sonication dispersion, and then the solutions were used for the combined triple treatment. After treatment for 24 h or 72 h, the viability of PANC-1 cells was measured using MTT assay. (TIF) [file pone.0201920.s005.tif]
